# Algae as an alternative source of protein in poultry diets for sustainable production and disease resistance: present status and future considerations

**DOI:** 10.3389/fvets.2024.1382163

**Published:** 2024-04-10

**Authors:** Ahmed A. A. Abdel-Wareth, Ayanna Nate Williams, Md Salahuddin, Sachin Gadekar, Jayant Lohakare

**Affiliations:** ^1^Department of Animal and Poultry Production, Faculty of Agriculture, South Valley University, Qena, Egypt; ^2^Poultry Center, Cooperative Agricultural Research Center, Prairie View A&M University, Prairie View, TX, United States; ^3^Algae Center of Excellence, Cooperative Agricultural Research Center, Prairie View A&M University, Prairie View, TX, United States

**Keywords:** algae, egg, microbiome, nutritional values, meat, sustainability, quality

## Abstract

Integrating algae into poultry diets offers a promising avenue for enhancing nutrition, boosting sustainability efforts, and potentially stimulating disease resistance. This comprehensive review delves into the essence, diversity, chemical composition, and nutritional merits of algae, spotlighting their emergence as innovative nutrient sources and health supplements for poultry. The growing interest in algae within poultry nutrition stems from their diverse nutritional profile, boasting a rich array of proteins, lipids, amino acids, vitamins, minerals, and antioxidants, thus positioning them as valuable feed constituents. A key highlight of incorporating both macroalgae and microalgae lies in their elevated protein content, with microalgae varieties like Spirulina and Chlorella exhibiting protein levels of up to 50–70%, outperforming traditional sources like soybean meal. This premium protein source not only furnishes vital amino acids crucial for muscular development and overall health in poultry but also serves as an exceptional reservoir of omega-3 fatty acids, notably eicosapentaenoic acid (EPA) and docosahexaenoic acid (DHA), presenting multiple health benefits for both poultry and consumers alike. Moreover, algae boast antioxidant properties attributed to bioactive compounds like phycocyanin and astaxanthin, mitigating oxidative stress and boosting the bird’s immune response, thereby fostering robust health and disease resilience. Incorporating macroalgae and microalgae into poultry diets yields positive impacts on performance metrics. Research evidence underscores the enhancement of growth rates, feed conversion ratios, carcass quality, and meat attributes in broilers, while in layers, supplementation promotes increased egg production, superior egg quality, and increased concentrations of beneficial nutrients such as omega-3 fatty acids. Furthermore, algae hold promise for mitigating the environmental footprint of poultry production, though significant outcomes from trials remain sporadic, necessitating further research to elucidate optimal dosages and blends for different algae species in poultry diets. Standardizing the composition of algae utilized in research is imperative, paving the way for potential applications in poultry nutrition as growth stimulants and substitutes for antibiotics. Nonetheless, a deeper understanding of dosage, combination, and mechanism of action through rigorous scientific investigation is key to unlocking algae’s full potential within poultry nutrition.

## 1 Background

Brown, green, and red macro- and micro-algae varieties are collectively referred to as “algae” in this context. Micro-and macro-algae could serve as novel sources of nutrients and health supplements for animals. Algae are a diverse class of plants that come in a variety of sizes, forms, and hues. Algae is a promising substitute protein that could eventually replace soybeans in poultry nutrition. These proteins may potentially improve the environment and unquestionably compete with soybeans in terms of protein quality. In addition, macroalgae and microalgae can be grown for ingredients and dietary supplements for animal and poultry feed, as well as carbohydrates, proteins, micronutrients, lipids, food additives, and cosmetics (1). Martins et al. (2) reported that microalgae have a unique composition of proteins, lipids, carbohydrates, minerals, vitamins, and bioactive compounds such as carotenoids. Algae are recommended as feed additives due to their high levels of macro-and micro-elements and ability to improve the growth performance, feed efficiency, and meat quality of broilers (3), which is primarily due to properties of polysaccharides that can increase the health and productivity of chickens. Feeding Spirulina to broiler chickens improved immunological measures, which improved cellular and humoral immunity as well as the formation of healthy gut microbiota (4). While existing literature consistently highlights microalgae as a rich source of bioactive compounds, their specific composition and subsequent impact on health outcomes—such as host immunity and intestinal integrity—require comprehensive investigation (5, 6). Macroalgae and microalgae have been shown to provide health benefits to poultry due to their anti-inflammatory and antioxidant capabilities (7–9). Algae are thought to be a potential prebiotic and may thus be used to improve digestive function, increase nutrient absorption, prevent antibiotic overuse, lessen the negative effects of antibiotics on wildlife microbiota, and reduce antimicrobial agent risk (10). Feeding freshwater green microalgae *Chlorella vulgaris* to broiler chickens at 1.55 g/kg increased growth performance and breast meat yield (11). *Spirulina platensis* at 0.25–1.0% in broiler feeds significantly enhanced growth performance including body weight, body weight gain, and feed conversion ratio (12). Dietary supplementation with 1% or 2% docosahexaenoic acid (DHA)-rich microalgae significantly improved carcass traits including thigh muscle, liver, spleen, antioxidant status, and abdominal fat in broilers (13). The improvements in carcass criteria and abdominal fat can be attributed to the favorable effects of DHA in microalgae on lipid utilization in serum as evidenced by lower serum cholesterol and triglyceride levels (13, 14). Recent reviews have underscored algae’s potential in poultry nutrition, spotlighting its nutritional benefits and health-enhancing properties (2, 15, 16). Despite these advances, significant gaps remain in fully understanding the specific health impacts of algae-based feeds, particularly their roles in enhancing immune function, gut health, and sustainability in poultry. Our analysis reveals persistent gaps, particularly in the meticulous mechanisms through which algae impact poultry health, such as their immunomodulatory effects, the capacity to optimize gut microbiota composition, and their pivotal role in fortifying gut integrity, thereby promoting sustainable productivity. The core objectives of our review are to encapsulate a comprehensive overview of algae and their derivatives, delving deep into the utilization of microalgae in chicken diets. We aim to illuminate the multifaceted benefits of algae, including their antioxidant and antimicrobial properties, their substantial impact on health status, productive performance, and the marked improvement in the quality of meat and eggs. By synthesizing the latest research findings and filling the identified research gaps, our review aspires to provide a novel perspective on the integral role of algae in advancing poultry nutrition and enhancing production efficiency, moving beyond the conventional paradigms of nutritional supplementation to a more holistic understanding of algae’s benefits in poultry diets with a focus on antioxidant, antimicrobial, health status, productive performance, and the quality of the meat and eggs.

## 2 Definition of algae

Algae refers to a diverse group of photosynthetic organisms that can be found in aquatic environments including freshwater and marine ecosystems. They are classified as simple non-flowering plants and are primarily divided into three groups: green algae, brown algae, and red algae. However, algae are not always classified as plants and can sometimes be classified as protists or even bacteria. Algae vary in size and complexity ranging from unicellular microscopic organisms to large multicellular seaweeds. They can be found in various colors from green to brown, red, and even golden. These organisms play a critical role in the environment and ecosystems. Algae are primary producers meaning they convert sunlight, water, and carbon dioxide into organic matter through the process of photosynthesis. This process generates oxygen as a byproduct contributing significantly to the Earth’s oxygen supply. Moreover, algae serve as the foundation of the food chain, furnishing essential nutrients and energy for other organisms such as fish, invertebrates, and even whales. More than 100,000 species of microalgae are classified into four groups: eukaryotic diatoms, green algae, golden algae, and blue-green algae (6). They are primarily autotrophic, utilizing carbon dioxide as their carbon source and sunlight as their energy source. However, certain heterotrophic microalgae utilize organic carbon instead of sunlight for energy. The latter can be easily cultivated in bioreactors and utilized to produce biomass. Microalgae can thrive in various environments, including coastal plains, deserts, and semiarid regions. As a result, the taxonomy associated with this group is complex and debatable among scientists. It consists of red (Rhodophyceae), brown (Phaeophycean), and green algae (Chlorophyceae). The very varying makeup of each form of algae is greatly influenced by species, habitat, growing conditions (such as temperature, and light exposure), and collection techniques. The term “algae” refers to a broad class of plants that includes seaweed and a variety of single-celled organisms. Algae are unisexual, simple, and frequently aquatic plants. Although algae lack genuine stems, roots, leaves, and vascular tissue, they do have chlorophyll. Microalgae have a unique composition of proteins, carbohydrates, lipids, vitamins, minerals, and bioactive substances such as carotenoids (17). Microalgae do not require fertile soil to develop, in contrast to land-based vegetation. The mix of macro-and micronutrients is determined by various parameters, including species, strain, growing circumstances, and biomass status. *Spirulina* (*Arthrospira*) *platensis* is a blue-green microalga renowned for its high-quality protein content. Among microalgae used in animal production, *Spirulina (Arthrospira* sp.*)*, *Chlorella* sp., and *Schizochytrium* sp. are frequently utilized. Numerous farmed finfish, shellfish, and other economically significant aquaculture species are produced in hatcheries using cultivated microalgae. In addition to these well-established uses of macro-and microalgae in aquaculture hatcheries, there is currently a push to incorporate algae into poultry diets. Many scientific advancements and commercial uses to date have centred on algae as a microfeed component that provides the recipient animal with specific beneficial characteristics as opposed to basic nutrients. In discussing the multifaceted roles of algae within agricultural contexts, particularly regarding animal and poultry health, it is critical to consider the adverse effects posed by certain toxic algae species. While algae can be a beneficial supplement in the diets of livestock and poultry, offering a rich source of nutrients, the presence of toxic algae species, such as some diatoms and dinoflagellates associated with red tide events, introduces significant risks (18, 19). These organisms can produce toxins that, when ingested, can lead to serious health issues and mortality in animals and birds. For instance, diatoms that produce domoic acid can cause neurological disorders and fatalities in marine wildlife, which, if transferred through the food chain, pose a threat to terrestrial livestock and poultry. Similarly, dinoflagellates responsible for red tides produce neurotoxins that can contaminate fish and shellfish (20), potentially affecting animals and humans that consume these affected resources. The implications of such toxic algae blooms are far-reaching, affecting not only the direct health of livestock and poultry but also the safety of food supplies and the broader agricultural economy. Thus, agricultural practices must include robust monitoring and management strategies for water quality and feed sources, aiming to mitigate the risks associated with toxic algae and ensure the health and safety of animal populations and, ultimately, human consumers.

## 3 Chemical and nutritional value of microalgae

There is an increasing need for macroalgal and microalgal meals on an international level, and people are consuming more algae for reasons other than nutrition and health. Algal-derived food products have been shown to have positive health impacts on humans and animals, although quantifying these advantages and any potential negative effects is still extremely challenging. Little information is known about the chemical compositions of various algae species, geographical regions, seasons, technology of drying, duration of storage and extraction process all of which have a significant impact on their nutritional value. Algal sources have a unique chemical makeup and nutritional value. Microalgae boast a distinctive composition encompassing proteins, carbohydrates, lipids, vitamins, minerals, and bioactive compounds, including carotenoids (2). Furthermore, microalgae serve as exceptional sources of omega-3 fatty acids, notably EPA and DHA, which offer numerous health benefits for both poultry and consumers. Despite their potential to reduce serum total cholesterol and low-density lipoprotein cholesterol, dietary intakes of high levels of EPA (C20:5n-3) and DHA (C22:6n-3) remain suboptimal in humans (21, 22). Low levels of algal biomass (1 to 5%) have been shown to improve DHA concentrations in poultry meat (23) as well as eggs (24, 25). Microalgae are also a good source of pigments, vitamins, minerals, and fatty acids (FA), particularly EPA and DHA, which have been shown to improve broiler meat quality (6). The ratio of micronutrients is regulated by several factors, including species, strain, growth conditions, and biomass status (17). The composition and yield of microalgae are impacted by changes in growing conditions. Microalgae is high in n-3 polyunsaturated fatty acids (PUFAs), carotenoids, B vitamins, and non-starch polysaccharides including beta-glucans (5). Individually, these chemicals have demonstrated health advantages linked with the anti-inflammatory and antioxidant capabilities of n-3 PUFAs and carotenoids, respectively (7–9). While published literature consistently identifies microalgae as a source of these compounds, their composition and subsequent impacts on health outcomes, such as host immunity and intestinal integrity, vary among genera and strains (5, 6). Algae are added to diets either directly as raw food or after processing, which concentrates any possible health benefits of certain extracts. Furthermore, microalgae are abundant in proteins, soluble fibers, polysaccharides, lipids, polyunsaturated fatty acids, pigments, vitamins, and minerals (26). The utilization of algae and its byproducts can effectively generate new food alternatives, yielding rich biomass suitable as substitutes for plant-based meat products, akin to tofu, tempeh, lentils, chickpeas, and almonds (27). Several full-and defatted (lipid-extracted) microalgae varieties exhibit moderate to high concentrations of crude protein (ranging from 14 to 61%), alongside a well-balanced profile of essential amino acids (%) including lysine (0.47–8.4), arginine (0.42–6.4), phenylalanine (0.36–5.0), threonine (0.42–4.8), histidine (0.20–2.0), methionine (0.33–2.3), tryptophan (0.13–2.1), and branched-chain amino acids such as leucine (0.59–8.8), isoleucine (0.13–3.8), and valine (0.47–5.5) (28). When following a plant-based diet, consumers should prioritize specific essential nutrients and/or foods, including plant-based meals rich in protein, vitamins (such as vitamin B12, and vitamin D), minerals (Ca), and essential omega-3 fatty acids (29). With research on the digestion and bioaccessibility of algal biomass in various food matrices, several recent studies reported in the literature have suggested using entire algae or algae extract for the creation of new foods. The chemical composition of dried Spirulina revealed that it contained around 60% total protein and all essential amino acids, with a total fat concentration of 7% (30), it is also high in vitamins, such as water-soluble vitamins C, B1, B2, B3, B6, B9, and B12, as well as fat-soluble vitamins A and E. Spirulina also has a high mineral content, including calcium, phosphorus, sodium, potassium, iron, chromium, copper, selenium, manganese, magnesium, and zinc (31). *Arthrospira platensis* and *Chlorella vulgaris* have a high crude protein content with a high biological value (32). The microalgae species also exhibit high total phenolic content ranging from 510 to 2,200 mg gallic acid equivalents (GAE) per kilogram, as well as total flavonoids ranging from 66 to 550 mg per kilogram (33). Additionally, microalgae contain notable concentrations (%) of calcium (0.33–2.8), phosphorus (0.65–0.76), sodium (3.2–3.9), potassium (0.89–1.7), and trace elements (mg/kg) such as iron (1,390–11,100), copper (18–102), and zinc (28–64) (28, 34). Algal food products are becoming more popular around the world as they have proven to be highly beneficial in diets and healthy nutrition (35). It could be stated that algae can be used in poultry nutrition due to their various nutritional benefits. Algae, particularly *Spirulina* sp. and *Chlorella* sp., are rich in protein. Including algae as a protein source in poultry diets can help meet the amino acid requirements for growth and development. Algae contain various essential nutrients, including vitamins, minerals, and fatty acids. These nutrients can contribute to overall poultry health and productivity.

## 4 Algae as antioxidants

**Figure 1:**
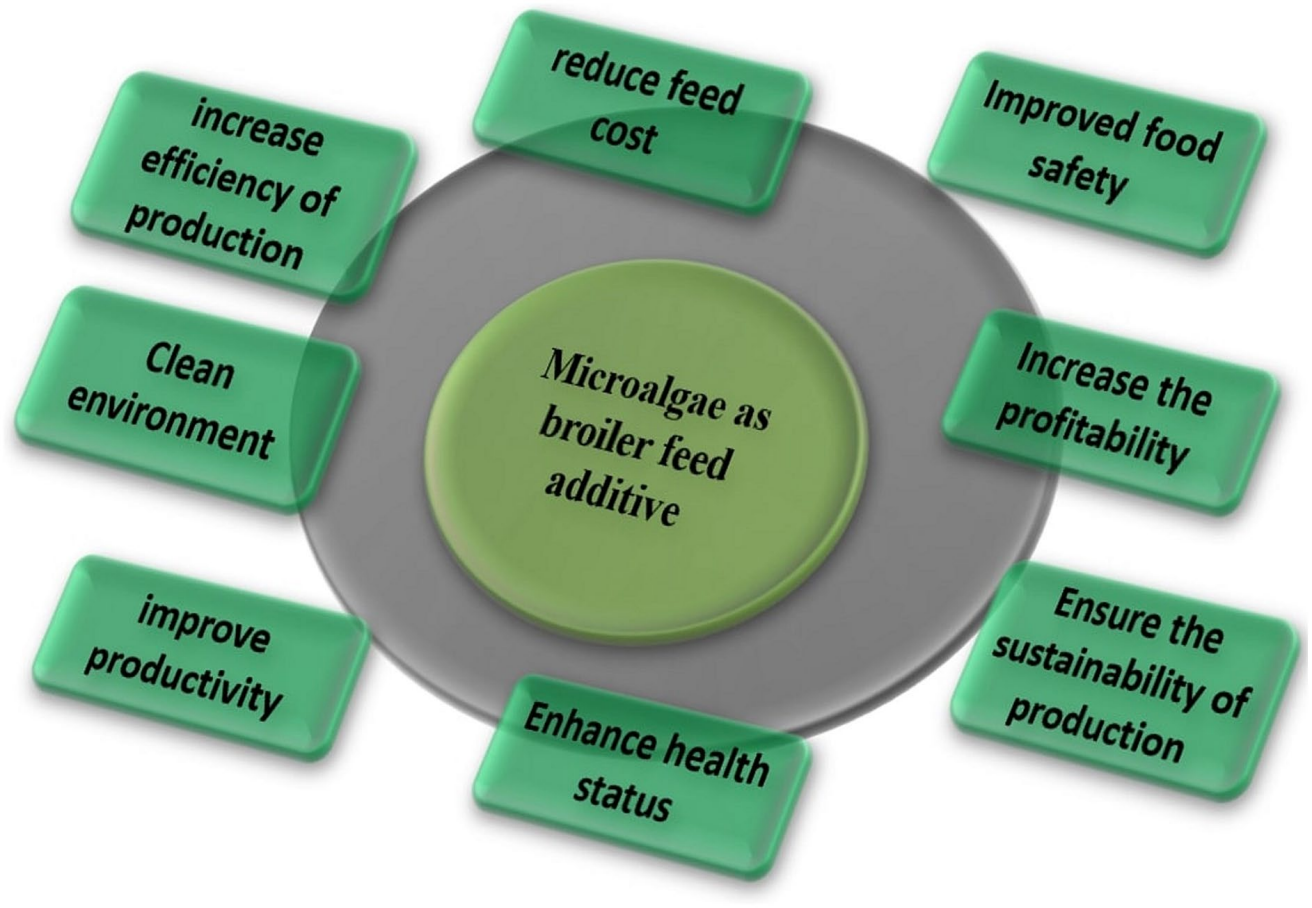
Microalgae have antioxidant, antibacterial, antiviral, and health benefits.

**Figure 2:**
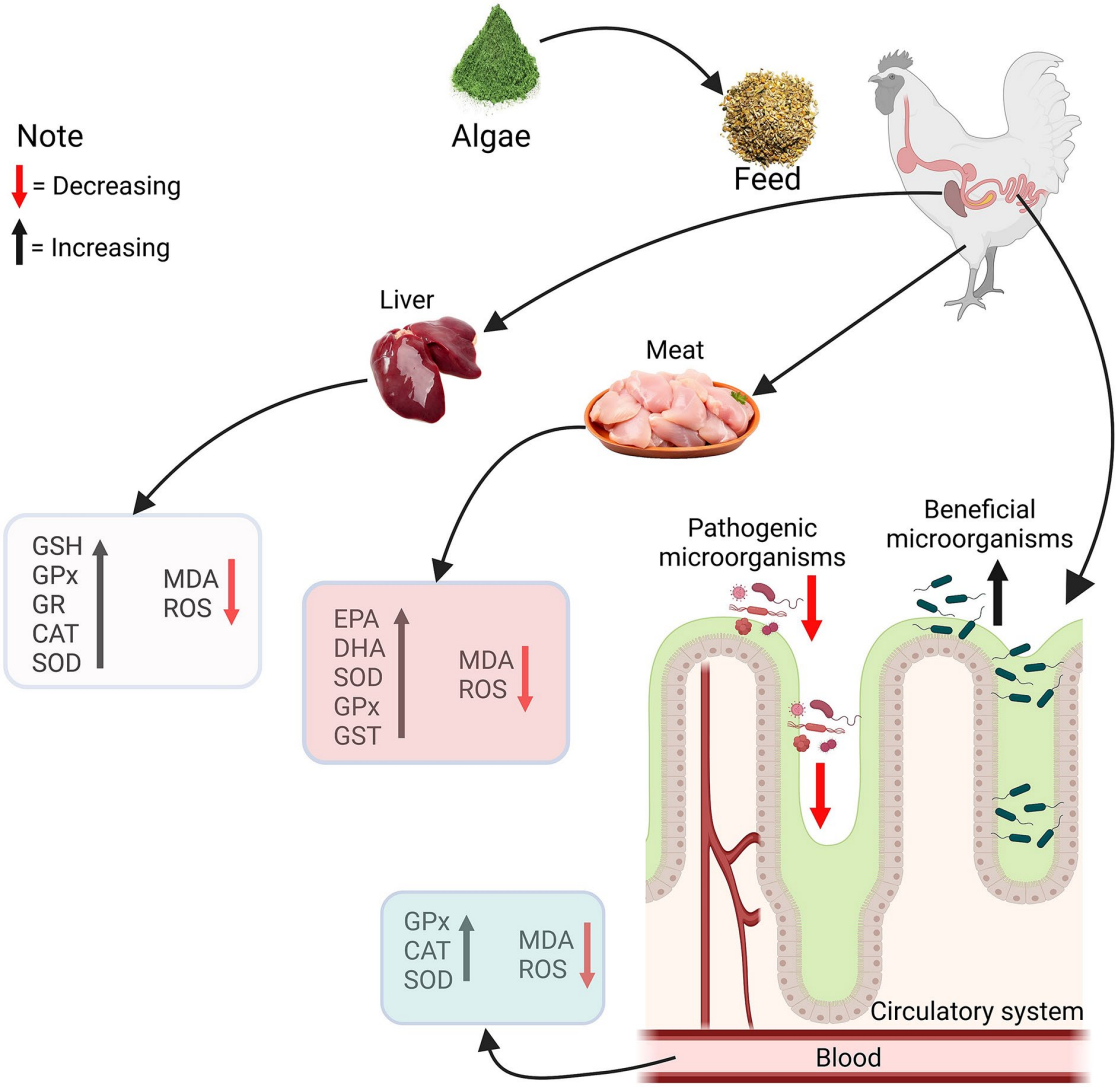
Schematic representation of the impact of algae supplementation on chicken health. The diagram illustrates the pathways through which algae enhance the antioxidant defense mechanisms within the liver and meat tissues, as well as their effects on the gut microbiota. Glutathione (GSH), Glutathione peroxidase (GPx), Gutathione reductase (GR), Catalase (CAT), Superoxide dismutase (SOD), Malondialdehyde (MDA), Reactive oxygen species (ROS), Eicosapentaenoic acid (EPA), Docosahexaenoic acid (DHA), Glutathione S-transferase (GST).

Algae contain antioxidants, such as carotenoids and tocopherols, which can help neutralize free radicals and reduce oxidative stress. Antioxidants may contribute to maintaining poultry health and preventing certain diseases. Microalgae have antioxidants, antibacterial, antiviral and health-benefit properties as shown in Figure 1. Microalgae have antioxidants that may play an essential role in protecting endogenous lipids from peroxidation and oxidation. The elimination of reactive oxygen species and the provision of endogenous and exogenous antioxidants regulate the body’s antioxidant status (13). Endogenous antioxidants include enzymes such as superoxide dismutase (SOD), glutathione (GSH), glutathione peroxidase (GPX) and glutathione reductase (GR). Broilers are prone to lipid peroxidation due to dietary and physiological factors, resulting in increased peroxidative metabolites. Feeding lipid-rich diets to birds may result in oxidative stress, which can damage DNA, bio-membrane lipids, and proteins, as well as a range of tissue impairments (36). Malondialdehyde (MDA) is a prominent byproduct of lipid peroxidation and a good indicator of oxidative stress (37). Figure 2 illustrates the pathways through which algae enhance the antioxidant defense mechanisms within the liver and meat tissues, as well as their antimicrobial effect on the gut microbiota. The supplementation of feed with algae leads to an increase in the levels of GSH, GPX, GR, catalase (CAT), and SOD in the liver, alongside a decrease in MDA and reactive oxygen species (ROS), indicating reduced oxidative stress. In meat, there is an elevation in EPA and DHA levels, as well as an increase in SOD, GPX, and GST activity, coupled with a reduction in MDA and ROS. In the circulatory system, an increase in GPX, CAT, and SOD is observed, along with a decrease in MDA and ROS. Additionally, the gut microbiota demonstrates a decrease in pathogenic microorganisms and an increase in beneficial microorganisms. The enzymatic inactivation of ROS in muscle tissue primarily relies on higher concentrations of SOD, CAT, and GPX. This is because SOD and CAT are antioxidant enzymes that directly interact with radical species, while GPX replenishes oxidized antioxidants (36). According to Sun et al. (38), supplementation with the highest dose of microalgae Astaxanthin enhances GSH, GPX, GR, and GST activities in the liver, breast, and thigh muscles of broiler chicks at 21 days of age. Furthermore, owing to the elevated DHA concentration in microalgae, birds fed microalgae exhibited increased levels of EPA, DHA, SOD, and total antioxidant capacity, along with a higher polyunsaturated fatty acid ratio, polyunsaturated fatty acid/saturated fatty acid ratio, and malondialdehyde in the breast and thigh muscles compared to the control group (13). Moreover, supplementing with spirulina was able to restore redox equilibrium because of its bioactive antioxidant components (39). Additionally, supplementing broiler chicken with spirulina under conditions of prolonged heat stress considerably increases levels of SOD and GPX while lowering MDA levels (40, 41). Floridoside is a red algal photosynthetic product that acts as a storage molecule, and antioxidant (42). Floridoside was found to have considerable antioxidative properties in clinical studies (43). SOD and GPX levels in broiler blood increased linearly in response to dietary spirulina at concentrations of 0.25, 0.5, 0.75, or 1.0% (44). Supplementation of *Haematococcus pluvialis* up to 0.004% in the diet increased oxygen radical-absorbance capacity, GSH, activities of GPX and SOD in the muscle (31). Furthermore, the inclusion of 10% of *S. platensis* in broiler diets improved antioxidant status (45). Despite being exposed to heat stress, supplemental dietary microalgae were accessible to the chicks and enhanced in their tissues, causing coordinated alterations in their endogenous antioxidant defense and meat quality (38). Microalgae have been shown to provide health benefits to broilers due to their antioxidant capabilities (7–9). Dietary supplementation with 1% or 2% DHA-rich microalgae significantly improved the antioxidant status of broiler meat (13). A variety of algae-derived chemicals exhibit antioxidant effects (46, 47). *Spirulina platensis* also contains phycocyanin, carotenoids, polysaccharides, and phenolic substances which have been demonstrated to have antioxidative properties (8). Spirulina has been found to promote health and immunity in broilers as well as diminish oxidative stress caused by heat stress (41).

Abdel-Moneim et al. (42) found that supplementing Spirulina species with selenium nanoparticles improved antioxidant activities and growth performance in heat-stressed broiler chicks. The modulatory effects of Spirulina antioxidation (48) may be related to carotenoids, B vitamins, n-3 polyunsaturated fatty acids, and non-starch polysaccharides such as beta-glucans in algae (4). Agustini et al. (40) found that dried Spirulina contains high bioactive compounds (i.e., phenolics, flavonoids, saponins, triterpenoids and steroids), which contribute to its high antioxidant activity. *Spirulina platensis* has antioxidant properties (49). Brown algae include a variety of bioactive chemicals with antioxidant activities (50), they are known to inhibit the hyaluronidase enzyme, which serves as a neuroprotectant for bone-related illnesses, as well as to limit the matrix metalloproteinase enzyme activity. Algae-active compounds, such as phycoerythrin, are now widely used in biomedicine due to their antioxidant properties (51). Supplementing diets with microalgae like *Haematococcus pluvialis* and *Spirulina platensis* upregulates antioxidant activities in muscle tissues, contributing to enhanced oxygen radical absorbance capacity and antioxidant enzyme activities (31). Additionally, supplementation with DHA-rich microalgae significantly improves broiler meat’s antioxidant status and reduces abdominal fat (13, 14). The beneficial effects on antioxidant status and abdominal fat contribute to improved bird immunity and fatty acid deposition in chicken meat (16). In conclusion, algae-derived antioxidants offer a promising opportunity for enhancing poultry health, supporting antioxidant defense mechanisms, and mitigating oxidative stress-induced ailments in broilers.

## 5 Algae as antimicrobials

Algae may contain prebiotic compounds that promote the growth of beneficial gut microorganisms. This can contribute to improved gut health and nutrient digestibility in poultry. Algae have a variety of therapeutic qualities, including antibacterial capabilities. Figure 2 illustrates the pathways through which algae enhance the antimicrobial defense mechanisms of the gut microbiota. The gut microbiota, a diverse community of microorganisms residing in our intestines, plays a crucial role in maintaining overall health. These microbes influence digestion, immune responses, and even impact systemic inflammation. Algae, often associated with aquatic environments, have recently gained attention for their potential health benefits, including their interaction with gut health. Furthermore, antibacterial activity pertains to compounds that either kill bacteria or inhibit their growth on a local level, all while remaining non-toxic to surrounding tissues. Immunity, on the other hand, refers to the body’s capacity to combat a specific disease, often achieved by either evading pathogenic bacteria or neutralizing the effects of their byproducts. Various chemicals derived from algae, such as polysaccharides, pigments, lectins, amino acids, and phenolic compounds, demonstrate antibacterial effects (45, 46).

Numerous *in vitro* studies have already confirmed the antibacterial activity of macro-and microalgae against pathogenic bacteria and fungi from rivers, and lakes. The algae produce an immense number of secondary metabolites that are effective against different kinds of harmful microorganisms. As a result, various *in vivo* studies were carried out to confirm the benefits of algae in the nutrition of poultry.

*Spirulina platensis* contains phycocyanin, carotenoids, polysaccharides, and phenolic substances which have been demonstrated to have immunostimulatory, antibacterial, and antiviral properties (52). Adding *Spirulina platensis* to broiler feed significantly improved the levels of specific gut bacteria like Ruminococcus, Oscillospira, Lactobacillus, Oscillobacter, Flavonifractor, and Colidextribacter, which are known for producing volatile fatty acids. This inclusion contributes to the better formation of healthy gut microbiota in chickens (4). Furthermore, algae supplementation was linked to improved gut health and systemic immune responses in the first seven days following Eimeria exposure (53). The improvements in intestinal integrity associated with algae were unique to Eimeria exposure, implying that algae may maintain protective benefits throughout time, particularly in pathogen-challenged situations (53). *Spirulina platensis* exhibits antiviral and antibacterial properties (49). Algae-active compounds, such as phycoerythrin, are now widely used in biomedicine due to their antibacterial and anti-inflammatory properties (51). Algae are thought to be a potential prebiotic and may thus be used to improve digestive function, and nutrient absorption, prevent antibiotic overuse, lessen the negative effects of antibiotics on wildlife microbiota, and reduce antimicrobial agent risk (10). *Chondrus crispus* and *Sarcodiotheca gaudichaudii* fed at a 2% dietary inclusion level significantly increased concentrations of the probiotics *Bifidobacterium longum*, *Lactobacillus acidophilus*, and *Streptococcus sali*var*ius* in the gut of laying hens (53). Frazzini et al. (54) reported that algae bioactive compounds can function as antioxidants and influence the proliferation of O138 *E. coli*. Fucoidan from *Turbinaria ornata* and *Sargassum polycystum* has been shown in previous studies to have strong antibacterial activity against bacterial pathogens such as *Vibrio harveyi*, *Staphylococcus* spp., *E. coli*, *Aeromonas hydrophila*, *Enterobacter* spp., *Pseudomonas aeruginosa*, *Streptococcus* spp., *Vibrio parahaemolyticus, Vibrio alginolyticus, Vibrio cholerae, Yersinia enterocolitica* and Proteus (55). Additionally, the addition of spirulina has been shown to have a positive impact on the gut microbial community, with an increase in *Lactobacillus* sp. and a decrease in *E. coli* populations (56). As the previous results, various *in vitro* and *in vivo* studies were carried out to confirm the benefits of algae as an antimicrobial in poultry. Algae inclusion in poultry diets can contribute to overall health and disease resistance. A healthy immune system, optimal nutrition, and a balanced microbiome are key components in preventing diseases.

## 6 Role of microalgae as an immune stimulant and in disease resistance

**Figure 3:**
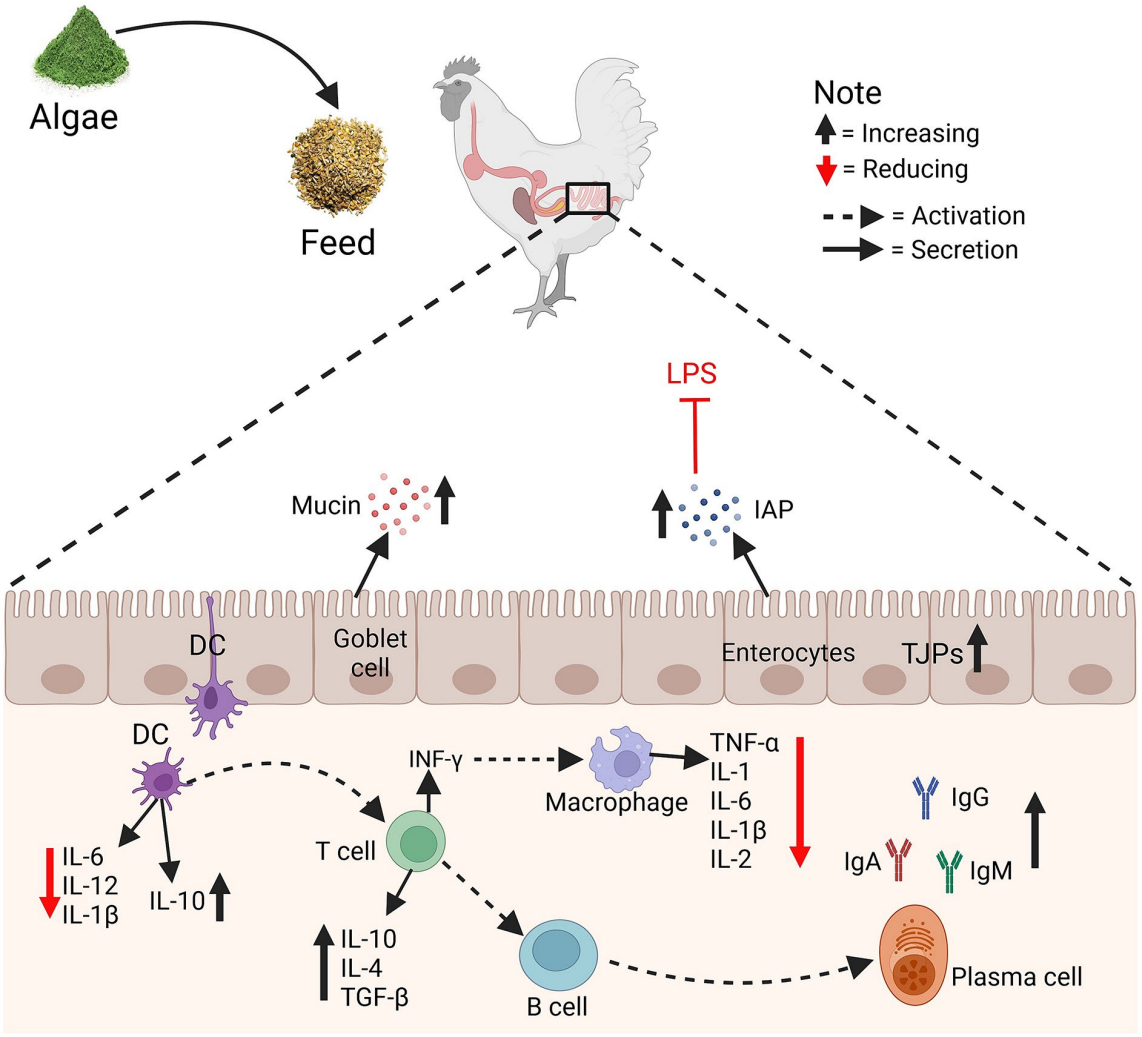
The effects of algae on gut immunity and integrity. Tight junctions (TJPs), Intestinal alkaline phosphatase (IAP), Lipopolysaccharides (LPS), and Dendritic cells (DC).

Some algae species have immunomodulatory properties that may positively influence the immune system of poultry. Enhanced immune function can contribute to disease resistance and overall health. Figure 3 illustrates the influence of algae supplementation in chicken feed on gut immunity and integrity. Algae intake enhances mucin production by goblet cells and strengthens tight junctions (TJPs) in enterocytes, thereby reinforcing the gut barrier. The algae also induce increased intestinal alkaline phosphatase (IAP) activity, which detoxifies lipopolysaccharides (LPS), reducing their pro-inflammatory impact. Dendritic cells (DC) shift towards an anti-inflammatory profile, decreasing the secretion of IL-6, IL-12, and IL-1β, and promoting the release of IL-10 and TGF-β. This environment encourages T cells to produce IL-10 and IL-4, further supporting an anti-inflammatory milieu. Macrophages exhibit reduced levels of TNF-α, IL-1, IL-6, and IL-2, signifying a lean towards a resolution phase of inflammation. B cells are triggered to increase IgA production, critical for mucosal immunity while modulating IgG and IgM levels. Immunity refers to the ability to fight a certain disease, particularly by avoiding pathogenic bacteria infections or counteracting the effects of its products. A variety of algae-derived chemicals (for example, polysaccharides, pigments, lectins, amino acids, and phenolic compounds) exhibit anticancer effects (45, 46). *Spirulina platensis* is a microalga with numerous medicinal characteristics, health advantages, and biological functions (57). *Spirulina platensis* also contains phycocyanin, carotenoids, polysaccharides, and phenolic substances which have been demonstrated to have immunostimulatory, hepatoprotective, and anti-inflammatory properties (47). Spirulina has been found to promote health and immunity in broilers (40). Feeding *Spirulina* sp. at 5% to diets of broiler chickens improved immunological measures, which improved cellular and humoral immunity as well as the formation of healthy gut microbiota (4). Though published research consistently points to microalgae as a source of these active compounds, their composition and consequent effects on health outcomes, such as host immunity and intestinal integrity (5, 6), are not reported. Microalgae has been shown to provide health benefits due to its anti-inflammatory capabilities in poultry (7–9). Microalgae have antioxidants, which may play an essential role in protecting endogenous lipids from peroxidation and oxidation. The elimination of ROS and the provision of endogenous and exogenous antioxidants regulate the body’s antioxidant status (13), which might improve the health and immune system of poultry. Clinical studies have revealed that the red algal photosynthetic product floridoside has strong anti-inflammatory and immunomodulatory effects (42). Furthermore, the inclusion of 10% of *S. platensis* in broiler diets improved health status and reduced circulatory inflammation of chickens (44). Despite being exposed to heat stress, supplemental dietary microalgae were accessible to the chicks and enhanced in their tissues, causing coordinated alterations in their endogenous antioxidant defense and meat quality (35). Abdel-Moneim et al. (42) reported that supplementing *Spirulina* species with selenium nanoparticles improved immunoglobulins and antibody titers against Newcastle disease. Comparatively, more focus has been placed on the immune-modulating properties of spirulina as a feed supplement in chicken that may enhance disease resistance and improve survival and growth rates, particularly under stressful conditions (4). Additionally, in the first seven days after exposure to Eimeria, algae supplementation was connected to enhanced gut health and systemic immune responses (58). *Spirulina platensis* demonstrates immunomodulatory characteristics (49). Brown algae are known to inhibit the hyaluronidase enzyme, which acts as a neuroprotectant for bone-related illnesses, as well as to limit the matrix metalloproteinase enzyme activity. These bioactive chemicals have been shown to have anti-inflammatory, anticancer, antidiabetic, antihypertensive, and antiallergic activities (50). Due to their anticarcinogenic, neuroprotective, and anti-inflammatory capabilities, algae-active substances like phycoerythrin are increasingly frequently employed in biomedicine (51). The microalgae elevated the intestinal integrity of broilers exposed to Eimeria, suggesting that algae may continue to provide protective effects over time, particularly in environments where pathogens are becoming an issue (59). Algae may be utilized to enhance digestion, boost nutrient absorption, avoid antibiotic misuse, lessen the harmful effects of antibiotics on wildlife microbiota, and decrease the risk of antimicrobial agents (10).

## 7 Algae for the sustainability of broiler production

**Figure 4:**
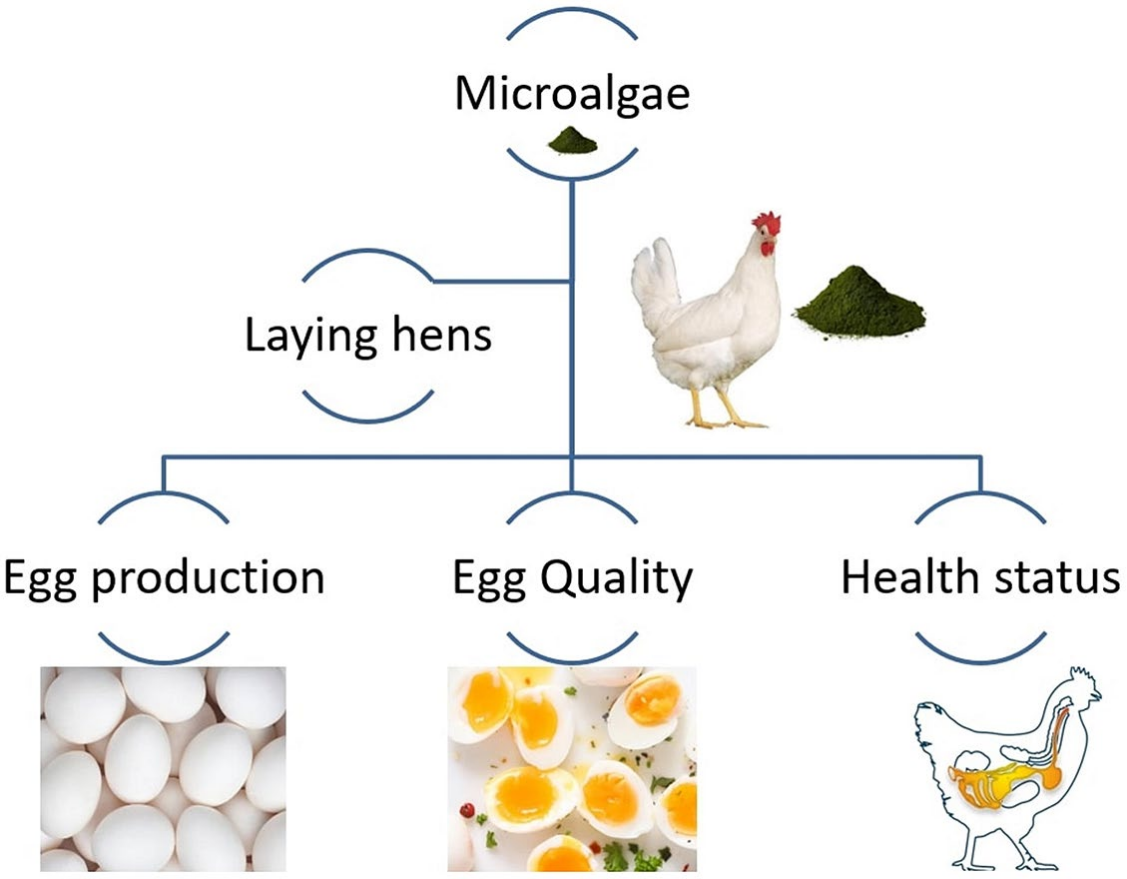
Microalgae as ecofriendly agents to improve production performance of broiler chickens.

Microalgae of various forms and sources are utilized to feed broiler chickens. Many feeding experiments have been performed to investigate the potential of various microalgal species as an alternative feed protein and their effects on the production performance and/or health status of broiler chickens. Research has shown that including microalgae in broiler diets can improve the production performance and meat quality attributes (Figure 4). Algae is recommended as feed additives due to their high levels of macro-and micro-elements and ability to improve the growth performance and feed efficiency of broilers (3), owing to the properties of seaweed polysaccharides that can increase the health and productivity of chickens. Table 1 shows a variety of feeding studies’ results for growth performance and meat yield when various microalgae were added to broiler diets.

**Table 1:** Effects of dietary supplementation with different microalgae on growth performance and meat criteria of broiler chickens.

Algae species	Levels	Effects	References
*Chlorella vulgaris, Spirulina platensis, or Amphora coffeaformis*	0.1%	All examined microalgae enhanced growth performance, decreased microbial growth in breast muscle, malondialdehyde and protein carbonyl levels, cooking loss, and aerobic plate count, and boosted superoxide dismutase activities in breast muscle.	El-Bahr et al. (11)
*Spirulina platensis*	0.25, 0.5, 0.75, or 1.0%	enhanced growth performance, carcass and meat yields, oxidative stability, and fatty acids modulation	Sharmin et al. (12)
*Schizochytrium*	3.7%	Increased body weight gain and feed conversion ratio as well as the fatty acid content of meat	Ribeiro et al. (60)
*Nannochloropsis oceanica*	2%	No effect on growth performance	Gatrell et al. (14)
*Spirulina platensis*	4 or 8%	No effect of Spirulina on performance and relative weights of internal organs. Pigmentation (yellowness) of muscles, skin, fat, and liver increased with an increasing dietary level of Spirulina	Toyomizu et al. (61)
*Spirulina platensis*	0.001, 0.01, 0.1, 1.0%	No effects of Spirulina on performance. Leghorn chicks in Spirulina dietary groups had increased total anti-SRBC titers; birds had increased phagocytic potential of macrophages and NK-cell activity	Qureshi et al. (62)
*Spirulina platensis*	6, 11, 16, or 21%	Dietary levels up to 16% algae resulted in a similar performance as in control group. The positive effect of algae inclusion was observed on the digestible methionine content in the diet	Evans et al. (63)
*Spirulina platensis*	0.5, 1.0, or 1.5%	A positive effect of 1% Spirulina on BWG, feed conversion ratio, and villus height	Shanmugapriya et al. (64)

Feeding microalgae (*Chlorella vulgaris*) to broiler chickens at 1.55 g/kg increased body weight gain by 8% and breast meat yield by 5% (11). *Spirulina platensis* at 0.25, 0.5, 0.75, or 1.0% in broiler feeds significantly enhanced growth performance including body weight, body weight gain, and feed conversion ratio (12), potentially due to the presence of bioactive substances and high amino acid digestibility (43). According to Ribeiro et al. (60), adding *Schizochytrium* to a broiler chicken diet at 3.7% significantly increased body weight and body weight gain compared to control. In broilers, fed Spirulina-enclosed diets with 5 or 10 g/kg feed, a significant increase in growth rate and improvement in feed conversion ratio were reported (56). Due to the high apparent metabolizable energy and high amino acid digestibility of the Spirulina-supplemented diet (65), as well as the detrimental effects of heat stress on intestinal morphology and feed intake, it is possible to partially justify the beneficial effects of Spirulina supplementation on broiler performance. Microalgae, including Arthrospira, commonly known as Spirulina, enhance the immune system of broilers, provide protection against various pathogens, and boost T-cell activity within the body (66). Furthermore, dietary supplementation with 1% or 2% DHA-rich microalgae significantly increased body weight gain and improved feed conversion of broilers (13). The evidence of increased growth performance connected with algae-derived compounds may be inconsistent. In many cases, algae incorporation did not affect broiler feed conversion ratio but improved body weight gain, indicating that changes in underlying physiology have no negative influence on bird performance (39). However, broiler chickens fed defatted green microalgae *Nannochloropsis oceanica* at 2% did not affect the body weight gain of broilers (14). Supplementing broiler diets with *Spirulina platensis* at high doses of 14 or 15% reduced body weight gain (67), possibly as a result of the low quality of the algal biomass used and/or the biomass’s tendency to gel at high concentrations, which is linked to a higher digesta viscosity. On these lines, Toyomizu et al. (61) reported that supplementation of *Spirulina platensis* at 4 or 8% did not reveal any appreciable variations in the growth performance or meat output. Supplementing the diet with microalgae at appropriate levels can enhance growth performance, increase relative carcass and organ weight, improve gut structure (villus height and crypt depth), and modulate ileal gene expression associated with immunity, gut barrier functions, antioxidant activities, and nutrient transportation (68). Birds, on the other hand, demonstrated no effects or decreased growth performance in the previous studies, which could be attributed to the high fiber (polysaccharide) content of algal biomass, and high concentrations of phenolic compounds (15).

Concerning carcass criteria, algae is recommended as feed additives due to their high levels of macro-and micro-elements and ability to improve broiler meat criteria (3). Broiler chickens fed freshwater green microalgae *Chlorella vulgaris* at a rate of 1.55 g/kg produced 5% more breast meat (11). According to Sharmin et al. (12), adding *Spirulina platensis* to broiler diets at 0.25 to 1.0% dramatically improved carcass metrics. This improvement may have been caused by the bioactive compounds and high amino acid digestibility (43). *Schizochytrium* at a concentration of 3.7% was added to the broiler chicken diet and it considerably boosted the meat carcass production compared to the control as reported by Ribeiro et al. (60). Dietary supplementation with 1% or 2% DHA-rich microalgae significantly improved carcass traits including thigh muscle%, liver%, spleen%, antioxidant status, and abdominal fat in broilers (13). The improvements in carcass criterion and abdominal fat can be attributed to the favorable effects of DHA in microalgae on lipid utilization in serum (14), as evidenced by lower serum cholesterol and triglyceride levels (15). The improved liver and spleen percentages as well as antioxidant status were advantageous to bird immunity and fatty acid deposition in chicken meat (16). In terms of meat quality, Sun et al. (38) reported that adding astaxanthin-rich *Haematococcus pluvialis* to a broiler diet increased the pH of the breast muscle and lowered the water-holding capacity (WHC) of the breast muscle compared to the control. By changing the expression of tenderness, WHC, and pH-related genes, supplemental dietary microalgae may have an impact on the physical and chemical properties of chicken (69). The WHC of muscle samples shows their ability to retain fluid during handling and processing. When *Schizochytrium*, *Aurantiochytrium*, and *N. oceanica* biomass were included in broiler diets, there were increases in the amount of EPA and DHA in meat compared to control-fed basal diets (70, 71). These enrichments were connected to reduced mRNA expression of cytochrome enzymes, higher mRNA expression of desaturases, fatty acid synthase, and elongases, and enhanced protein production of acetyl-CoA carboxylase (70, 71). There were no negative effects of dietary supplementation with 1% or 2% DHA-rich microalgae on thigh and breast pH value, lightness, redness, yellowness, or drip loss of broilers compared with the control diet (13). El-Bahr et al. (11) found that the inclusion of microalgae (*Chlorella vulgaris*, *Spirulina platensis*, and *Amphora coffeaformis*) at a concentration of 0.1% resulted in reduced microbial growth in breast muscle, as well as decreased levels of malondialdehyde and protein carbonyls, reduced cooking loss, and lowered aerobic plate count. Additionally, it was observed that the inclusion of microalgae increased SOD activities in breast muscle. Regarding nutritional and physiological characteristics, broilers fed microalgae showed improved concentration of serum total cholesterol, glucose, total protein, albumin, urea, and creatinine of broilers compared with control (13). Serum glucose is an important source of energy for animals and can promote the growth of body tissues, whereas serum albumin and total protein concentrations reflect the functions of protein synthesis in broiler liver, which may be associated with animal growth and physiological status (72). Indeed, DHA from microalgae led to reductions in serum cholesterol and triglycerides, while increasing the levels of high-density lipoprotein cholesterol (HDL-C) (73). This effect is attributed to DHA’s ability to decrease triglyceride levels by reducing hepatic synthesis of very low-density lipoprotein (VLDL) (74). The improved blood composition observed in birds may potentially mitigate the risk of cardiovascular disease in humans who consume chicken meat from broilers fed microalgae-based diets (13). Moreover, utilizing algae as feed aligns with sustainability goals, as algae cultivation typically requires less land, water, and fertilizers compared to traditional protein sources like soybeans. This underscores the potential of algae as a sustainable feed ingredient, reducing the environmental footprint of poultry production.

## 8 Algae for sustainable egg production and quality

**Figure 5:**
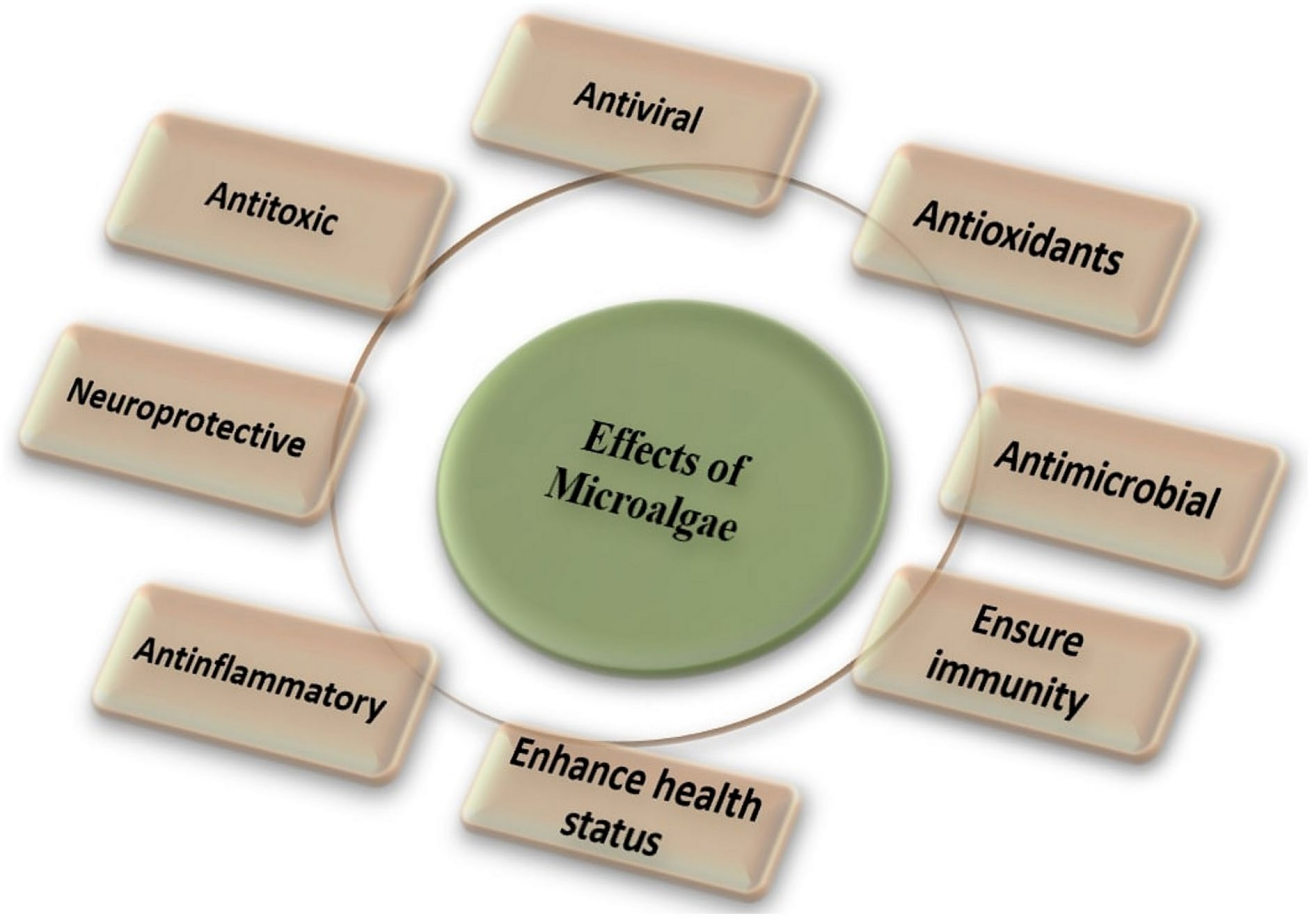
Microalgae as eco-friendly agents to improve laying hens’ production and egg quality.

Egg production and quality are essential factors in the poultry industry. Farmers are constantly looking for ways to improve these aspects to meet the growing demand for high-quality eggs. One promising solution lies in the use of microalgae. Microalgae of various types and origins are used to feed laying hens. Many feeding experiments have been conducted to evaluate the possibility of various microalgal species as an alternative feed protein and their impact on laying hen production and/or health. In layers, microalgae supplementation can enhance egg production, egg quality, and health status (Figure 5). They are packed with nutrients such as protein, vitamins, minerals, and essential fatty acids making them a valuable addition to poultry feed. Microalgae are rich in essential nutrients that are beneficial for the overall health of the hens. When incorporated into their diet it provides a concentrated source of proteins, vitamins, and minerals. This improves the hens’ nutritional intake resulting in better egg production.

About microalgae’s role in enhancing egg production, studies have shown that feeding hens with microalgae can lead to an increase in egg size. The findings of various feeding studies on the production and quality of eggs after the addition of various microalgae to layer diets are shown in Table 2. The nutrients present in microalgae can stimulate the ovaries and promote the development of larger eggs. This is particularly valuable for egg producers aiming to meet specific size requirements in the market. For example, the average egg weight and egg mass increased dramatically after receiving spirulina-based diets (29). Kalia and Lei (39) reported that supplementation of *Schizochytrium* (0.5–1%), *Spirulina platensis* (1.5–2.5%), and *Chlorella vulgaris* (2.5–7.5%) to laying hens diets increased egg production rate (from 85 to 91%) and egg weight (from 62.8 to 64.3 g). On the other hand, Herber and Van Elswyk (80) found that hen’s fed a diet supplemented with 4.8% marine algae had lower egg output than hens fed a control diet without algae. The laying egg production, egg mass, and egg weight improved by adding spirulina to layer diets at a concentration of 0.3% (76).

**Table 2:** Effects of dietary supplementation with different microalgae on egg production and quality of laying hens.

Algae species	Levels	Effects	References
*Chlorella vulgaris*	1.2%	Improved egg weight, feed conversion ratio, sell quality, yolk color, yolk lutein and zeaxanthin, as well as oxidative stability of yolk lipids of fresh and stored eggs.	Englmaierova and Skrivan (75)
*Schizochytrium*	0.5 and 1%	Increased albumin weight, eggshell weight, and eggshell thickness	Englmaierova and Skrivan (75)
*Spirulina platensis*	0.3%	It can be utilized as a natural feed supplement with functional properties that improve laying performance, egg quality, and hepatoprotective action.	Selim et al. (76)
*Schizochytrium*	0.5 and 1%	Increased egg weight and production as well as egg quality	Kalia and Lei (39)
*Spirulina platensis*	1.5 and 2.5%	Increased egg production and quality	Kalia and Lei (39)
*Chlorella vulgaris*	2.5 and 7.5%	Increased egg production and quality	Kalia and Lei (39)
*Nannochloropsis oceanica*	2%	No effects on growth performance	Gatrell et al. (14)
*Spirulina platensis*	4 or 8%	Improved cholesterol and triglycerides in eggs	Toyomizu et al. (61)
*Chlorella vulgaris*	2%	Enhanced yolk carotenoids lutein, B-carotene, and zeaxanthin concentration and egg color score in chickens, but it lowered yolk weight.	Kotrbacek et al. (12)
*Chlorella vulgaris*	1%	Improved egg production, yolk color, and lutein concentration in serum, liver, and developing oocytes. 0.2% lutein-fortified Chlorella boosted egg weight, yolk color, and lutein.	An et al. (77)
*Spirulina platensis*	1.5, 3.0, 6.0, or 12.0%	Improved growth performance, egg production and quality	Ross and Dominy (78)
*Chlorella vulgaris*	0.1 or 0.2%	Improved egg production, performance, egg yolk color, Haugh units, and microflora profile including intestinal acid bacteria, cecal population biomass.	Zheng et al. (79)

Concerning egg quality, the value of eggs to consumers and the economics of chicken farming are both impacted by egg quality. The strength and quality of eggshells are crucial in preventing breakage during handling and transportation. Microalgae supplements have been found to enhance eggshell quality by providing essential nutrients like calcium and phosphorus. These nutrients play a vital role in the formation and maintenance of strong eggshells reducing the risk of egg breakage. The average egg weight, yolk color, eggshell strength, and iron content all increased dramatically after receiving spirulina-based diets (81). In addition, the levels of cholesterol and triglycerides in eggs were significantly decreased. Use of Spirulina (2.0–2.5% of the diet) as chicken feed has already demonstrated improved yolk color in eggs (74). The yolk color is an essential quality parameter for consumers and plays a significant role in their perception of egg quality. Microalgae contain natural pigments such as carotenoids which contribute to the vibrant yellow-orange yolk color. Including microalgae in the hens’ diet can result in eggs with a richer and more appealing yolk color. In addition, the levels of cholesterol and triglycerides in eggs were significantly decreased by supplementation of spirulina in laying hens diet compared to control (81). Supplementation of algal biomass (1 to 5%) to laying hens diet has been shown to improve DHA concentrations in eggs (21, 22). Omega-3 fatty acids are highly beneficial for human health and their presence in eggs is a desirable quality. Microalgae are an excellent source of omega-3 fatty acids such as EPA and DHA. Therefore, feeding hens with microalgae can increase the omega-3 content in the eggs providing consumers with a healthier option. Zeller et al. (23) pointed out that *Schizochytrium* sp. contained carotenoids, specifically canthaxanthin, and beta-carotene, which are major sources of yolk pigmentation. *Schizochytrium* at 0.5–1% and *Chlorella* sp. at 1.2% supplemented to laying hen diets increased albumin weight, eggshell weight, and eggshell thickness (75). The egg yolk’s total EPA, DHA, and omega-3 fatty acid levels were linearly (*p* < 0.01) enriched by the defatted *Nannochloropsis oceanica* microalgae (82). The 15% defatted diatom microalgal biomass (DFA) diet increased egg albumen weight and height when compared to the 7.5% DFA and control diets (83). Although the exact mechanism by which microalgae altered egg albumin weight or eggshell weight and thickness has not been determined, it may be related to the plentiful amino acids and minerals in the microalgal biomass.

The chicken’s health status is improved by feeding microalgae to chickens. The hepatoprotective activity of hens was enhanced by adding spirulina to layer diets at a concentration of 0.3% (74). Algae is recommended as a feed additive due to its high levels of macro-and micro-elements and ability to influence the health of birds and the quality of eggs (3), which can increase the productivity of chickens as well as the quality of their eggs due to the properties of seaweed polysaccharides. Incorporating microalgae into poultry feed can have significant benefits for both egg production and quality. The nutrient-rich profile of microalgae improves overall hen health resulting in increased egg production and larger egg sizes. Additionally, it enhances eggshell strength, yolk color, and omega-3 fatty acid content. As the demand for high-quality eggs continues to rise, the use of microalgae in poultry feed offers a sustainable and effective solution for egg producers.

## 9 Algae effects on microbiome and histomorphology in poultry

*Ascophyllum nodosum* exhibited effectiveness in the lower gastrointestinal tract (GIT) by altering intestinal pH, histomorphology, bursa, and cecal relative weights, suggesting fermentation in the GIT by beneficial microorganisms (84). Incorporating *P. palmata* (1.8%) into broiler diets increased levels of beneficial bacteria (*Lactobacillus*) in the ileum, serum IgA, as well as the breadth, height, and surface area of ileal villi in chickens (85). The inclusion of *P. palmata* at 0.15% in the diet led to improvements in diarrhea scores and the fecal microbiome, significantly reducing the relative abundance of pathogenic bacteria like *E. coli* while increasing that of beneficial bacteria such as *Lactobacillus* (86). Supplementation with seaweed boosted the population of beneficial gut bacteria such as *Bifidobacterium longum* (4- to 14-fold) and *Streptococcus salivarius* (4- to 15-fold) while reducing the incidence of *Clostridium perfringens* (87). Moreover, incorporating seaweeds into poultry feeds enhances gut microbiota by remaining predominantly undigested in the lower gastrointestinal tract (GIT), thereby serving as substrates for bacterial fermentation (88). The prebiotic-like properties of red and brown seaweeds influence the metabolic activities of beneficial microflora and decrease the prevalence of harmful bacteria (89). Additionally, a carbohydrate fraction derived from the red seaweed *Gracilaria persica* displayed direct antimicrobial effects against six bacterial pathogens, including *Staphylococcus aureus*, *E. coli*, *Methicillin-resistant Staphylococcus aureus*, *Salmonella typhimurium*, *Pseudomonas aeruginosa*, and *Aeromonas hydrophila*, while also inducing a humoral immune response against sheep red blood cells (56). Water extracts of red seaweeds *Gelidium latifolium*, *Hypnea musciformis*, *Jania rubens*, *Jania* spp., and *Laurencia obtusa* exhibited potent *in vitro* antimicrobial activity against Gram-negative pathogenic bacteria such as *E. coli*, *Klebsiella* spp., and *P. aeruginosa* (90). Furthermore, chickens fed *Laminaria japonica* at 1, 3, and 5% levels inhibited *E. coli* counts while increasing *Lactobacillus* counts in ceca (91). At both dosages, *Ascophyllum nodosum* (0.05 and 0.1%) reduced *Campylobacter jejuni* levels in the caecum of chicks at 10 days of age (92). *Sarcodiotheca gaudichaudii* at 1 and 2% concentrations in the diets of Lohmann Lite laying hens prevented the deleterious effects of *Salmonella enteritidis* infection (93). Previous research has shown that some red seaweeds contain quorum-sensing inhibitors, such as brominated furanones, which can block bacterial biofilm formation and the regulation of flagellar and virulence genes, resulting in bacterial growth inhibition (94, 95). Karimi (85) reported that *Palmaria palmata* (1.8%) increased villus height, width, villus surface area, and mucosal depth, as well as chicken plasma immunoglobulin (IgA and IgG). *Chondrus crispus* added to layer diets at 0.5, 1 and 2% increases villus height, width, villus surface area, and mucosal depth in ceca weight (96). Inclusion of *U. prolifera* at 1, 2, and 3% improved immunological function, and the number of beneficial bacteria, including *Bifidobacterium* and *Lactobacillus*, was significantly greater in the feces of laying hens compared to control groups, indicating improved animal health (97). Ethanolic extracts of several seaweeds were found to synergistically reduce bacterial growth by reducing efflux pump activity (98). An aqueous extract of brown and red seaweeds at 400 and 800 g/mL, in combination with sub-lethal dosages of tetracycline (1 g/mL and 1.63 g/mL), inhibited bacterial growth completely, comparable to full potency tetracycline (23 g/mL) (99). Microalgae supplementation in chicken diets increased immune function, gut integrity, and gut microbiota which improved nutrient digestion and production performance (62–64, 78, 86, 100, 101). Liu et al. (101) observed that dietary algae-derived polysaccharides (ADP) improved intestinal health, and the serum D-lactic acid concentration was reduced by dietary supplementation of 1,000 mg/kg, 2,500 mg/kg, and 7,000 mg/kg ADP on day 21 in broilers. As per the findings of Qureshi et al. (62), broiler chicks that were fed diets containing 1% Spirulina exhibited enhanced phytohaemagglutinin-mediated lymphocyte proliferation and increased phagocytic activity of macrophages compared to control., Replacing antibiotic growth promoters with *Chlorella* sp. improved performance, immunological indices, and the intestinal microflora population (102). Fermented *Chlorella vulgaris* improved lactic quality, and microflora profile, including intestinal acid bacteria cecal population, according to Zheng et al. (79). Extensive research has explored novel feed additives and unconventional ingredients in poultry nutrition to optimize nutrition, enhance productive performance, improve health status, stimulate immunity, and enhance egg and meat quality in chickens (103–108). This comprehensive approach considers the entire diet, environmental factors, and management techniques to achieve optimal poultry health and performance (77, 109–112). Future directions may necessitate closer collaboration among researchers, the poultry industry, and feed manufacturers to translate scientific findings into practical applications. This collaboration could accelerate the adoption of algae-based nutrition strategies in the poultry sector.

## 10 Eco-friendly algae production for poultry nutrition

Exploring sustainable agricultural practices, the innovative and eco-friendly production of algae using nutrient-rich effluents emerges as a promising frontier. The development of an innovative method for cultivating algae, specifically for species such as *Tetraselmis suecica* and *Dunaliella salina*, recognized for their significant nutrient content, introduces a sustainable approach to algae farming (113, 114). The cultivation of algae typically involves two main popular approaches: open-pond systems and closed-loop systems (115). Open pond systems utilize large outdoor ponds where algae are cultivated using natural sunlight and defined nutrient-rich media or effluents. These ponds require careful management of environmental conditions such as temperature, pH, CO_2_ and nutrient levels to ensure optimal algal growth. Closed-loop algae systems, on the other hand, employ controlled environments such as photobioreactors. These systems provide precise control over environmental parameters, allowing for consistent and efficient algae cultivation. There are considerable differences between these two systems as far as capital as well as operational costs of production are concerned. Efforts to optimize algae cultivation for poultry nutrition have led to the development of tailored cultivation techniques. These techniques focus on maximizing biomass productivity and nutrient content while minimizing resource inputs and environmental impact (15). For example, some companies utilize modular cultivation units that can be easily scaled up or down to accommodate varying production needs and operational constraints. By utilizing waste nutrients for algae production, this method embodies the principles of a circular economy, transforming potential environmental liabilities into valuable agricultural inputs. Highlighting the eco-friendly production of algae not only emphasizes its role as a sustainable feed component but also aligns with global sustainability objectives. Furthermore, the commercial viability of eco-friendly algae production as a poultry feed supplement is increasingly recognized by industry stakeholders. Such companies adopt advanced cultivation technologies, nutrient optimization strategies, and integrated production systems to produce high-quality algae biomass specific for poultry nutrition. However, the journey towards fully integrating these algae into poultry diets necessitates comprehensive research to evaluate their health benefits, optimize cultivation techniques further, and assess their economic feasibility within the poultry industry. Through dedicated exploration of these aspects, the poultry sector can advance towards more environmentally friendly and sustainable production systems, contributing to the well-being of poultry and the environment.

## 11 Future directions in research and development of using algae in poultry nutrition

Research and development in the field of using algae in poultry nutrition holds immense potential for the future of the poultry industry. Algae, including microalgae and seaweeds, offer a rich source of nutrients that can potentially revolutionize poultry diets, leading to improved growth performance, enhanced nutrient utilization, and overall health benefits for chickens. Moving forward, it is essential to delve deeper into the nutritional composition of various algae species to fully recognize their potential benefits for poultry. Researchers should focus on identifying and characterizing specific bioactive substances present in algae that have the greatest impact on poultry health and performance. This data and knowledge will inform the development of customized feed formulations optimized for different stages of poultry production. Additionally, future studies should aim to determine the optimal inclusion rates of algae in chicken diets at various production phases, including starter, grower, and finisher stages. Understanding how different levels of algae supplements influence growth performance, feed efficiency, and meat quality will be critical for optimizing poultry nutrition strategies. It is important to investigate the potential of algae-derived bioactive compounds as feed additives with beneficial effects on poultry health. Research needs to explore how these compounds contribute to disease resistance, gastrointestinal health, and immune function in chickens, paving the way for innovative approaches to enhancing poultry health. In addition to nutritional benefits, researchers need to assess the environmental sustainability of incorporating algae into poultry diets. This includes evaluating the carbon footprint, waste production, and resource usage associated with algae cultivation and processing. Finding ways to minimize environmental impact while maximizing nutritional benefits will be essential for promoting sustainable poultry production practices. To promote local sourcing and reduce reliance on conventional feed ingredients, studies should investigate the feasibility of using locally grown or obtained algae in poultry diets. This could involve exploring different cultivation methods and assessing the nutritional quality and cost-effectiveness of locally sourced algae as feed constituents. Understanding the effects of algae on the poultry gut microbiota is another crucial area for future research. Investigating how algae supplementation influences microbial diversity, nutrient absorption, and overall gut health in chickens will provide valuable insights into the mechanisms underlying the observed benefits of algae-based nutrition. Economic analysis is also needed to determine the cost-effectiveness of incorporating algae into commercial poultry production. Assessing the economic benefits, including improved growth rates, feed efficiency, and reduced medication use, will help poultry producers make informed decisions about adopting algae-based nutrition strategies. Moreover, efforts should be made to obtain regulatory approvals for the use of specific algae species in poultry diets and establish guidelines for their safe and effective incorporation into commercial feeds. Collaborative partnerships between researchers, feed manufacturers, and poultry producers will be essential for translating scientific findings into practical applications and accelerating the adoption of algae-based nutrition strategies in the poultry industry. By embracing cutting-edge technologies and interdisciplinary collaboration, the future of algae in poultry nutrition holds tremendous potential for advancing the sustainability, efficiency, and profitability of poultry production systems.

## 12 Conclusion

In conclusion, incorporating macroalgae and microalgae into poultry feed represents a significant advancement in optimizing poultry nutrition. The rich nutritional profile of algae offers a plethora of benefits, spanning from improving growth performance and egg production to enhancing the quality of poultry products such as meat and eggs. Algae’s abundance of proteins, lipids, vitamins, and minerals provides an all-inclusive approach to enhancing broiler and hen health. One of the most compelling aspects of algae-based nutrition lies in its potential to influence the gut microbiome of poultry positively. By promoting microbial diversity and the growth of beneficial bacteria, algae contribute to enhanced digestive health and nutrient absorption in chickens. This aspect necessitates further exploration through in-depth studies clarifying the intricate interactions between algae-derived compounds and the gut microbiota. Moreover, algae’s role as a growth stimulant and a viable alternative to antibiotics underscores its significance in poultry production. However, to fully capitalize on the benefits of algae, it is crucial to conduct systematic studies to determine the optimal dosage and combinations for different stages of poultry production. Additionally, elucidating the precise mechanisms of action underlying algae’s effects on poultry health and performance will be crucial for informed decision-making in feed formulation. As we attempt sustainable practices in poultry nutrition, algae emerge as a promising solution to address the challenges posed by conventional additives. Their potential to reduce reliance on antibiotics while enhancing productivity and product quality signifies a paradigm shift in poultry feed formulation. By promoting interdisciplinary collaborations and investing in cutting-edge research, we can unlock the full potential of algae-based nutrition, helping in a new era of sustainable and health-conscious poultry production.

## Author contributions

AAAA-W: Conceptualization, Data curation, Resources, Validation, Visualization, Writing – original draft, Writing – review & editing. AW: Conceptualization, Data curation, Methodology, Validation, Writing – original draft, Writing – review & editing. MS: Conceptualization, Data curation, Methodology, Validation, Visualization, Writing – original draft, Writing – review & editing. SG: Methodology, Writing – original draft, Writing – review & editing. JL: Conceptualization, Data curation, Funding acquisition, Investigation, Methodology, Project administration, Resources, Supervision, Validation, Visualization, Writing – original draft, Writing – review & editing.
